# The effect of calibration factors and recovery coefficients on ^177^Lu SPECT activity quantification accuracy: a Monte Carlo study

**DOI:** 10.1186/s40658-021-00365-8

**Published:** 2021-03-18

**Authors:** Keamogetswe Ramonaheng, Johannes A. van Staden, Hanlie du Raan

**Affiliations:** Department of Medical Physics, Faculty of Health Sciences, University of the Free State, PO Box 339, Bloemfontein, 9300 South Africa

**Keywords:** ^177^Lu, Quantification, Monte Carlo, SIMIND, Calibration factor, Recovery coefficients

## Abstract

**Background:**

Different gamma camera calibration factor (CF) geometries have been proposed to convert SPECT data into units of activity concentration. However, no consensus has been reached on a standardised geometry. The CF is dependent on the selected geometry and is further affected by partial volume effects. This study investigated the effect of two CF geometries and their corresponding recovery coefficients (RCs) on the quantification accuracy of ^177^Lu SPECT images using Monte Carlo simulations.

**Methods:**

The CF geometries investigated were (i) a radioactive-sphere surrounded by non-radioactive water (sphere-CF) and (ii) a cylindrical phantom uniformly filled with radioactive water (cylinder-CF). Recovery coefficients were obtained using the sphere-CF and cylinder-CF, yielding the sphere-RC and cylinder-RC values, respectively, for partial volume correction (PVC). The quantification accuracy was evaluated using four different-sized spheres (15.6–65.4 ml) and a kidney model with known activity concentrations inside a cylindrical, torso and patient phantom. Images were reconstructed with the 3D OS-EM algorithm incorporating attenuation, scatter and detector-response corrections. Segmentation was performed using the physical size and a small cylindrical volume inside the cylinder for the sphere-CF and cylinder-CF, respectively.

**Results:**

The sphere quantification error (without PVC) was better for the sphere-CF (≤ − 5.54%) compared to the cylinder-CF (≤ − 20.90%), attributed to the similar geometry of the quantified and CF spheres. Partial volume correction yielded comparable results for the sphere-CF-RC (≤ 3.47%) and cylinder-CF-RC (≤ 3.53%). The accuracy of the kidney quantification was poorer (≤ 22.34%) for the sphere-CF without PVC compared to the cylinder-CF (≤ 2.44%). With PVC, the kidney quantification results improved and compared well for the sphere-CF-RC (≤ 3.50%) and the cylinder-CF-RC (≤ 3.45%).

**Conclusion:**

The study demonstrated that upon careful selection of CF-RC combinations, comparable quantification errors (≤ 3.53%) were obtained between the sphere-CF-RC and cylinder-CF-RC, when all corrections were applied.

## Background

Lutetium-177 (^177^Lu) has become widely used for targeted radionuclide therapy in nuclear medicine [1]. This is due to its favourable decay characteristics as both a therapeutic and an imaging agent [2]. In addition, it is produced with high specific activity and exhibits reliable labelling of peptides used for tumour targeting [3, 4]. ^177^Lu has gained favour in the clinical application of peptide receptor radionuclide therapy (PRRT) with ^177^Lu-DOTATATE for the treatment of patients with late-stage metastatic neuroendocrine tumours [5–9]. The kidneys have been identified as the dose-limiting organs for ^177^Lu-DOTATATE PRRT [10]. A significant correlation was found between the tumour-absorbed dose and tumour reduction [11]. Therefore, dosimetry should be extended to include both the kidneys and tumour sites to provide a satisfactory understanding of PRRT treatment. There is a large variation in kidney and tumour response among patients following PRRT [12, 13]. This would facilitate the application of accurate patient-specific dosimetry for PRRT treatment planning, as is the norm in external beam radiotherapy.

Accurate dosimetry is strongly dependent on the accuracy with which activity quantification can be achieved. The imaging and quantification process is not ideal and therefore has been investigated and refined for many years [14–17]. There are factors inherent in the imaging process that degrade the images from an ideal representation of the imaged object. These factors include photon attenuation and scattering, collimator blurring, partial volume effects (PVEs) and the reconstruction algorithms [18]. SPECT/CT images have been included in many dosimetry protocols for ^177^Lu activity quantification due to their superior quantitative accuracy compared to planar images [19, 20]. However, the effects of the above-mentioned degrading factors complicate SPECT imaging. Therefore, attempts to compensate for these factors have to be made to improve the accuracy of the quantified activity.

The 3D ordered subset expectation maximization (OS-EM) iterative reconstruction algorithm [21] can include modelling of the physical characteristics of the imaging process. These may include compensation for photon attenuation, photon scatter and collimator detector response (CDR). This algorithm results in reconstructed images with better image quality, improved quantitative accuracy and less prone to artefacts than analytical methods such as filtered back projection [22–24]. The 3D OS-EM reconstruction algorithm has become a standard algorithm with most clinical SPECT processing units; it is highly recommended and commonly used to obtain improved quantitative SPECT data [25]. An important consideration when using the above-mentioned 3D OS-EM reconstruction algorithm is the optimum number of updates which is defined as the product of the number of subsets and iterations. The choice for the number of iterations used in patient studies is a trade-off between the image noise level and improved activity quantitative accuracy, where the latter improves with an increasing number of iterations [26]. For quantification purposes, the criteria for the optimum number of 3D OS-EM updates have been accepted as the convergence where 90% of the activity has been recovered [26]. Complex reconstruction algorithms incorporating more corrections require a larger number of iterations to reach convergence, and larger objects have been shown to converge faster than smaller objects [27]. The percentage root mean square (%RMS) has been used to assess the noise levels in objects of interest [28–30]. It is recommended that phantom or Monte Carlo (MC) simulation studies be used to investigate the optimum number of 3D OS-EM updates needed to provide adequate convergence for accurate activity quantification [16]. The optimization is unique to a specific SPECT system and associated reconstruction algorithm as well as the imaging geometry.

The X-ray CT in hybrid SPECT/CT systems has provided an automated way to compensate for object attenuation. The availability of the co-registered SPECT and CT data has made routine attenuation compensation practical and easy to implement. Multiple window scatter compensation methods such as the dual energy window (DEW) and the triple energy window (TEW) methods are available with most gamma camera vendors, and these methods can be used to apply scatter correction during or post-reconstruction. However, model-based scatter correction methods, such as effective source scatter estimation (ESSE), model the scatter function more accurately and thus better resemble the true scatter distribution [31]. The ESSE method estimates the degrading scatter effect from a point source at various depths behind a slab of material (mimicking tissue) using MC simulations to pre-calculate a set of scatter kernels. The MC simulation methods are also used to model the geometric response, septal penetration and septal scatter response functions of the gamma camera from a point source in air. For SPECT imaging purposes, these response functions are incorporated during reconstruction to improve spatial resolution. The volume of interest (VOI) definition for object delineation is also an important consideration for quantification of SPECT images, and no standardised method has yet been identified. CT data has been used in a number of clinical and phantom studies for object delineation [17, 32]. The impact of errors due to misdefinition (variability in organ delineation) and misregistration (between emission and CT data) on SPECT and planar quantification accuracy has been investigated [33]. The VOI definition is affected by PVEs, which has a larger impact on smaller objects with a size smaller than three times the system spatial resolution (3 × FWHM) [34]. The PVE is related to the gamma camera’s limited spatial resolution and can be compensated for by including a CDR correction during the iterative reconstruction process. A consequence of the PVE is that the image counts from a photon originating from a point will contribute not only to a single voxel but also to neighbouring voxels. This is known as spill-out of counts and is widely seen in tumour imaging and reported to result in underestimation of the quantified activity distribution [35]. Conversely, spill-in of counts from surrounding radioactive objects is also observed resulting in an overestimation of the quantified activity. If this spill-in and spill-out of counts at the edges of objects is not compensated for, it will result in biased quantification results [18]. Recovery coefficients (RCs) for partial volume correction (PVC) can easily be determined in phantom studies where true activity and object size can be measured [36]. CT-based RCs have been successfully used for PVC in ^177^Lu SPECT activity quantification in a torso phantom [15] and extended to 3D-printed kidney phantoms [32]. Another important consideration of PVE is image sampling. Due to the limited voxel size, a combination of objects having different activity concentrations may contribute to a specific voxel count. Thus, a small shift in the delineated region can result in a significant variation in counts. The extent of the variation can be limited by using smaller voxel sizes [37].

SPECT was traditionally regarded as a non-quantitative imaging modality, unlike its counterpart PET. In both modalities, advances in hybrid systems include automation of compensation methods for photon attenuation, scatter and limited spatial resolution in a unified manner using iterative reconstruction algorithms. In general, SPECT images are obtained in units of image counts, unlike PET reconstructed images, which have units of tissue radioactive concentration (kBq.cm^−3^). Thus, a calibration factor (CF) has to be applied to obtain SPECT reconstructed images in units of radioactive concentration. It is only recently, with modern SPECT/CT systems, that reconstructed SPECT images are routinely provided in units of radioactive concentration. An example of such a SPECT/CT system is the Siemens Symbia Intevo™ scanner (Siemens Healthineers, Germany), which incorporates a point source traceable to a secondary standard laboratory for calibration purposes and conversion of the reconstructed SPECT images to activity concentration.

The system sensitivity, as defined by the National Electrical Manufacturers Association (NEMA) [38], is conventionally used as a CF in SPECT images to convert the quantified counts into units of activity [39–41]. It is generally accepted that the CF, with a source geometry that incorporates photon attenuation and scatter properties in the acquisition, reduces the effects of imperfect scatter and attenuation corrections [26]. Different geometries have been reported for obtaining CFs for ^177^Lu SPECT quantitative imaging [42]. These authors investigated four geometries to obtain CFs which included a point source in air, a sphere in air, a sphere in non-radioactive water and a 20 cm diameter cylinder uniformly filled with ^177^LuCl_3_. The point source in air and the sphere in water yielded the worst and best results respectively for the activity quantification in an anthropomorphic torso phantom. The sphere in a non-radioactive background was reported as suitable for activity quantification of ^177^Lu SPECT data using the 208 keV photopeak. Superior quantification accuracy has been reported for SPECT imaging with a CF obtained using a sphere in a radioactive background (sphere-to-background ratio of 6:1) compared to that obtained using a cylindrical phantom uniformly filled with ^131^I [27]. The authors also reported that the sphere CF yielded more stable results than a point source in air, which is highly sensitive to the selected VOI size. Due to down-scatter and septal penetration from high-energy photons into the photopeak window for isotopes such as ^131^I and ^188^Re, Zhao et al. [43] suggested that a CF obtained from a planar point source must include scatter correction for these isotopes, and to a lesser extent for the ^177^Lu 208 keV photopeak. In addition, the study showed that CFs obtained using spheres in a non-radioactive background may overestimate the CF by 10% attributed to the underestimation of the TEW scatter approximation and accentuated further by the attenuation correction during reconstruction.

The disparities between ^177^Lu SPECT activity quantification accuracy reported by different authors for various phantoms, as well as their data acquisition and processing protocols, have been summarised in the MIRD pamphlet 26 [16]. The MIRD pamphlet 26 also documents guidelines for ^177^Lu SPECT quantitative imaging established using the Simulating Medical Imaging Nuclear Detectors (SIMIND) MC program [44]. However, this document does not report the quantification accuracy or error obtained with the recommended methods. Each clinic should thus determine the quantification error relevant for their chosen setup and method. There is increasing evidence supporting the need for personalised dosimetry based on SPECT quantitative information. The attempt has been hindered by the lack of standardised methods, which include determining the gamma camera CF. As indicated in the above studies, there is no consensus with regard to the CF geometry. Wevrett et al. [45] demonstrated the feasibility of using simple phantom geometries to standardise gamma camera calibrations for activity quantification of ^177^Lu and ^131^I. These phantom geometries included a sphere placed centrally in air and in water as well as at an offset of 8.6 cm in water. The authors concluded that none of their geometries were sufficient to be used individually and recommended that a mean value obtained from the different geometries should be incorporated. They also suggested that the characterisation of the change in the CF with lesion volume was necessary due to the PVE and have proposed that the CF and RC should be combined in a mean calibration coefficient. This approach may require that a large range of CF values be determined from different geometries and tabulated. In addition, the CF would be affected by the VOI definition and further affected by the segmentation method used to determine the RC. In another publication, Wevrett et al. [46] assessed the feasibility of carrying out an international inter-comparison of European hospitals to determine the consistency of the CFs used at these sites. In the exercise, dual compartment spherical sources of known activity concentration and volume were sent to seven hospitals and acquired in a water-filled Jaszczak phantom. The hospitals acquired and processed the data using their own choice of methods. The authors reported that no single method reported by the different hospitals yielded a significantly improved accuracy. Further research has to be carried out to investigate the uncertainties associated with determining a suitable CF for ^177^Lu SPECT quantitative imaging.

This study aimed to investigate the effect of two CF geometries and their corresponding RCs on the quantification accuracy of ^177^Lu SPECT images using MC simulations. The CFs were obtained from (i) a radioactive sphere surrounded by non-radioactive water, termed sphere-CF, and (ii) a cylindrical phantom uniformly filled with radioactive water, termed a cylinder-CF. Two sets of RC curves were generated as a function of object size, using the two CFs. The first RC curve was constructed using the sphere-CF and the second RC curve was created using the cylinder-CF; the two curves were used to obtain the sphere-RC and cylinder-RC values, respectively. The effects of the sphere-CF-RC and cylinder-CF-RC combinations, on the quantification accuracy of ^177^Lu activity derived from SPECT images, were evaluated using three phantom geometries.

## Materials and methods

**Fig. 1:**
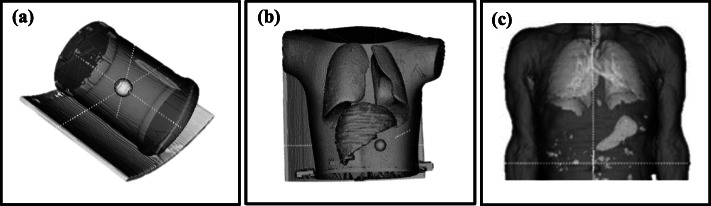
Segmented images of the **a** cylindrical, **b** torso and **c** patient phantoms

The SIMIND MC program (SIMIND version 6.1.2) was used in this study. Its ability to successfully mimic ^177^Lu SPECT images with a model of a Siemens Symbia T16 hybrid SPECT/CT (Symbia T16) (Siemens Medical Solutions, Inc. Hoffman Estates, IL., USA) dual-head gamma camera was validated in a previous study [47]. The validation included planar tests stipulated for gamma camera performance criteria following the NEMA recommendations [38]. The differences between the experimental and simulated values of three particular validation tests are significant to report herein. These three planar tests, namely the intrinsic energy resolution (%), the system spatial resolution (FWHM) and the system sensitivity (cps/MBq), had percentage differences of − 3.1%, 4.3% and − 3.0% between the experimental and simulated values, respectively. The two last parameters’ data was obtained in a 20% energy window centered over the 208 keV photopeak. Details describing the validation tests and the gamma camera physical parameters are defined in the above-mentioned publication. Three voxelized phantoms, as shown in Fig. 1, were used in this study. They were created by segmenting the CT images of these phantoms with the software ITK-snap (version 3.2.0) [48] as described in detail by Ramonaheng et al. [47]. The three phantoms shown in Fig. 1 were (a) a cylindrical phantom with a segmented volume of 9900 ml; (b) an RSD Alderson torso phantom (Radiological Support Devices Inc, USA) which was segmented to separate the lung inserts (combined segmented volume of 2090 ml), the liver insert (1090 ml) and the remainder of the phantom (2095 ml); and (c) a randomly selected SPECT/CT patient study anonymously obtained from the Symbia T16 patient database, approved by our institution’s ethics committee. For the patient phantom, the liver (1590 ml), lungs (2270 ml), spleen (160 ml), left (159 ml) and right (169 ml) kidneys, as well as the remaining volume of the patient (14,100 ml), were segmented. Each of the voxelized phantoms was appropriately equipped with spherical inserts using ITK-snap to mimic tumours.

The simulations were set up for ^177^Lu using a medium-energy (ME) collimator with a 20% energy window centred over the 208 keV photopeak (187.2–228.8 keV) [19, 49–51]. For all SPECT simulations, the phantoms were placed in the centre of the field of view and projection data were simulated over 360° with equally spaced projections using a non-circular orbit. The phantom to detector distance was determined for each projection angle using a density map to mimic auto-contour detection of the phantom outline [47]. All simulations were conducted with a high number of photon histories to ensure data sets with low simulation noise. Sixty (60) projections with an equivalent of 45 s acquisition time were simulated and the image data were stored in a 128 × 128 matrix with a pixel size of 4.8 × 4.8 mm [16, 52].

Our study comprised of two phases. The *first phase* of the study focused on three essential steps required to optimize the reconstruction parameters and the quantification process. The steps consisted of (i) optimizing the 3D OS-EM reconstruction algorithm, (ii) establishing the CFs and (iii) determining the RCs. The *second phase* of the study evaluated the quantification accuracy, reported as the quantification error, achieved with the steps established in the initial phase by quantifying spherical inserts in the three above-mentioned phantoms. The kidney quantification in the patient phantom was also evaluated. The simulations of the phantoms comprised of simulated projection images and result files describing the simulation protocol. A 3D OS-EM algorithm was used for image reconstruction and employed CT-based attenuation correction, ESSE scatter correction [31, 53] and CDR correction. The CDR correction included modelling of the intrinsic and geometric response as well as the septal penetration and septal scatter. All the reconstructed data were analysed using the public domain software Amide [54]. Spherical volumes corresponding to the physical dimensions of the spheres were used to delineate all the spheres. This method of delineation is analogous to a clinical scenario whereby co-registered CT images are used to define the target boundaries for quantitative SPECT images [15, 55, 56]. The mean counts from the fractional voxels were used to calculate the total counts from these VOIs.

### Quantification steps

For the first phase of the study, each of the phantom geometries in the quantification steps was simulated with a uniform ^177^Lu concentration of 3.04 MBq/ml. This is comparable to concentrations reported in the literature for similar investigations [57]. Different sphere sizes were simulated individually in the centre of the phantom to avoid spill-in of nearby spheres.

#### 3D OS-EM optimization

To determine the optimal number of 3D OS-EM updates required for convergence (where 90% of the true activity is recovered), SPECT images of a cylindrical phantom equipped with radioactive spheres with volumes (and their corresponding diameters) of 4.2 ml (2.0 cm), 14.1 ml (3.0 cm) and 65.4 ml (5.0 cm) in a warm background were simulated. To further investigate the effect of the sphere-to-background ratio on the convergence, each sphere was simulated using sphere-to-background ratios of 6:1 and 13:1 [55, 57, 58]. The reconstruction was performed as described above and multiple 3D OS-EM updates (ranging from 24 to 204) were used. As the spheres’ quantified activity is not yet known, the total counts for each sphere size were normalised to the maximum number of counts obtained at 204 updates for each particular VOI and expressed as percentage recovery. The aforementioned percentage recovery for each sphere was plotted as a function of the number of updates. The optimization process was repeated with a clinical geometry by simulating the left and right kidneys of the patient. Similarly, the kidney-to-background ratios and concentrations were equivalent to those used for the spheres. The convergence, needed for high reconstruction accuracy for both the sphere and kidneys, was defined as the 90% recovery [26]. The noise levels in the sphere and kidney volumes obtained for the different updates were calculated using Eq. 1.

1 $$ \%\mathrm{RMS}=100\times \frac{\mathrm{SD}}{\mathrm{mean}} $$

where RMS, mean and SD are the root mean square, the mean counts per voxel and the standard deviation of the reconstructed counts per voxel obtained from the target VOIs; namely the spheres and the kidneys. Although noise is typically evaluated by calculating the %RMS of the counts in a uniform area (29–30). In this study, it was however used to assess the noise level changes within the entire target VOIs as a function of the number of updates. In both cases of the spheres and kidneys, the VOIs were defined according to their physical dimensions described during the segmentation process (‘Materials and methods’ section). The sphere VOIs were defined as described previously, while the kidneys’ physical volumes were delineated as described subsequently in the ‘Evaluation of quantification accuracy’ section. The noise was assessed using the target objects’ physical volume, which provided a consistent delineation method that could be applied to evaluate the quantification accuracy.

#### Calibration factors

The sphere-CF was calculated using a simulation of a 65.4 ml (5.0 cm) radioactive sphere placed in the cylindrical phantom (Fig. 1a) with a non-radioactive background. The sphere diameter was selected to be more than three times the system spatial resolution to limit the influence of PVE on the accuracy of activity quantification. The planar system spatial resolution for the ^177^Lu 208 keV photopeak using a ME collimator was determined in our previous study, resulting in a FWHM of 1.1 cm [47]. The cylinder-CF was calculated using a simulation of the above-mentioned cylindrical phantom filled with a uniform activity distribution. Both geometries offered simple scatter and attenuation properties that could be used to minimise the effect of imperfect corrections. Considering that ^177^Lu is a costly therapeutic isotope, with a relatively long physical half-life of 6.7 days, the non-radioactive background of the sphere-CF offered a more practical alternative that could easily be implemented in clinical practice compared to the cylinder-CF. The CFs were calculated using the general formula shown in Eq. 2.

2 $$ \mathrm{CF}\ \left(\mathrm{cps}/\mathrm{MBq}\right)=\frac{\mathrm{CR}\ \left(\mathrm{cps}\right)/V\ \left(\mathrm{ml}\right)}{\mathrm{TC}\ \left(\mathrm{MBq}/\mathrm{ml}\right)\ } $$

where CF is the calibration factor, CR is the count rate, *V* is the volume of the VOI and TC is the true concentration obtained from the SIMIND result file. For the cylinder-CF, the cylindrical VOI was defined in a uniform area in the centre of the image to exclude edge effects, as recommended by D’Arienzo et al. [42]. The VOI for the sphere-CF corresponded to the physical dimensions of the sphere. The cylinder-CF was taken as the reference to calculate the percentage difference between the two CFs.

#### Recovery coefficients

The RCs for partial volume compensation were obtained from simulated SPECT images of the cylindrical phantom uniformly filled with ^177^Lu and radioactive spheres of eight varying sizes below and above the known system spatial resolution, with known concentration. The sphere volumes (and diameters) used were 2.4 ml (1.5 cm), 4.2 ml (2.0 cm), 8.2 ml (2.5 cm), 14.1 ml (3.0 cm), 22.4 ml (3.5 cm), 33.5 ml (4.0 cm), 47.7 ml (4.5 cm) and 65.4 ml (5.0 cm) with a sphere-to-background ratio of 6:1. The concentration was calculated using the sphere-CF and the cylinder-CF and expressed as a fraction of the true concentration (Eq. 3) to determine the RC of the sphere and cylinder respectively.

3 $$ \mathrm{RC}=\frac{C_{\mathrm{SPECT}}}{C_{\mathrm{true}}} $$

where *RC* is the recovery coefficient and *C*_SPECT_ and *C*_true_ are the SPECT estimated and true activity concentrations (MBq/ml) in the spheres, respectively. The values of the coefficients from the curve function shown in Eq. 4 were determined by fitting the mono-exponential function to the above-mentioned sphere and cylinder RC data to generate the sphere-RC and cylinder-RC values used for PVC.

4 $$ y=a-b\left({e}^{- cx}\right) $$

where *y* indicates the required RC value, *x* is the sphere diameter and *a*, *b*, and *c* are the fitting constants. The application of sphere-based RC curves (look-up tables) for PVC of the kidneys based solely on volume dependence has been reported to be non-optimal for ^177^Lu SPECT/CT image quantification [32]. This was due to the differences found between the renal RCs and sphere-RCs. The authors suggested the replacement of sphere-based RCs with geometry specific RC look-up tables for the kidneys. Therefore, instead of applying a volume-dependent RC value based on the fitted functions of the spheres, we opted to calculate the RC values for the sphere-RC and cylinder-RC using Eq. 3 directly. The generation of geometry specific look-up tables using kidneys of different volumes was beyond the scope of this study and the calculation of the kidney RC values in the above-mentioned manner seemed to be the better choice. The sphere-RC and cylinder-RC values, for the different size spheres and the kidneys used for PVC in the subsequent sections, were tabulated with their percentage differences.

### Evaluation of quantification accuracy

The second phase of the study evaluated the ^177^Lu SPECT quantification error in the three phantoms. The objective of the first phantom (cylindrical phantom) was to determine the ^177^Lu quantitative error of the spherical inserts in a phantom that characterised a simple cylindrical geometry and a homogeneous attenuating medium. The second phantom (torso phantom) represented a more complex geometry as well as a non-homogeneous attenuating medium. The third phantom extended the quantification error to a clinically realistic patient phantom. All the phantoms were simulated four times with the inclusion of one of the spheres of volume: 15.6 ml (3.1 cm), 24.4 ml (3.6 cm), 33.5 ml (4.0 cm) and 65.4 ml (5.0 cm) for each simulation, respectively. The sphere concentrations were 2.2 MBq/ml with a sphere-to-background ratio of 13:1. The sphere-to-background ratios selected for the three phantom simulations were different from those simulated for the RC (6:1) to avoid biased results in the determination of the quantification error. Each sphere was simulated in the centre of the cylindrical phantom as well as in the abdomen next to the liver for the torso and patient phantoms. The relative concentration in the various structures found in these two phantoms was defined to reflect typical activity distribution of ^177^Lu-DOTATATE [52, 59]. The torso phantom had lung and liver concentrations of 0.34 MBq/ml and 0.51 MBq/ml, respectively (similar to the patient phantom), with a background concentration of 0.17 MBq/ml. The spleen concentration was 0.52 MB/ml for the patient phantom, with corresponding kidney and background concentrations of 0.45 MBq/ml and 0.02 MBq/ml, respectively. It is important to note that all the segmented low-dose CT images were generated with a 5-mm slice thickness using a smooth reconstruction kernel (B08) to create the simulated phantoms. Therefore, the kidneys were segmented as whole left and right kidneys, encompassing the cortex, medulla and to a lesser extent the renal pelvis. The 3D iso-contour feature of Amide was used to delineate the kidney volumes. All areas above and below the minimum and maximum threshold values were chosen to result in each kidney’s equivalent volume as obtained from the simulation results. The VOI used for kidney delineation corresponded to the true (known) volumes established from the segmented kidneys, which included the whole left and right kidneys as described above. The kidney-to-background ratio of 23:1 for the patient phantom reflected a 24-h uptake for a ^177^Lu-DOTATATE pharmacokinetic study [52].

Two sets of quantification results were obtained for each of the phantoms. Firstly, the counts were converted to concentration using the sphere-CF without PVC to generate the sphere-CF quantification error, and subsequently corrected for partial volume using the corresponding sphere-RC to generate a sphere-CF-RC quantification error. Secondly, the counts were converted to concentration using the cylinder-CF without PVC to obtain the cylinder-CF results and the cylinder-RC applied for PVC, resulting in the cylinder-CF-RC data. The quantification error was calculated as the percentage difference between the true concentration (true (MBq/ml)) used as input into SIMIND and the quantitative SPECT estimated concentration (SPECT (MBq/ml)) calculated from the images (Eq. 5).

5 $$ \mathrm{Quantification}\ \mathrm{error}\ \left(\%\right)=\frac{\mathrm{SPECT}\ \left(\mathrm{MBq}/\mathrm{ml}\right)-\mathrm{True}\ \left(\mathrm{MBq}/\mathrm{ml}\right)\ }{\mathrm{True}\ \left(\mathrm{MBq}/\mathrm{ml}\right)}\times 100 $$

## Results

### Quantification steps

#### 3D OS-EM optimization

**Fig. 2:**
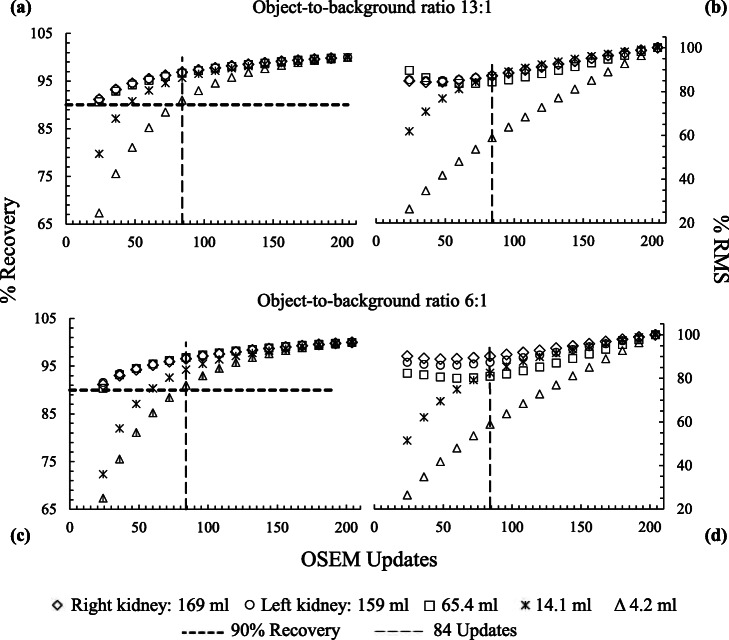
**a** Percentage counts recovered and **b** percentage root mean square (%RMS) obtained from reconstructed images as a function of 3D OS-EM updates for different size spheres simulated inside a cylindrical phantom, as well as for the right and left kidney of a patient phantom with an object-to-background ratio of 13:1. **c** Percentage counts recovered and **d** %RMS calculations were repeated for simulations with an object-to-background ratio of 6:1. Reconstruction included CT-based attenuation correction, effective source scatter estimation(ESSE) scatter correction and collimator detector response (CDR) compensation

The effect of the number of 3D OS-EM updates on the percentage recovery and the %RMS is illustrated in Fig. 2. The values in Fig. 2 were calculated from the reconstructed ^177^Lu SPECT images of three different-sized spheres simulated in a cylindrical phantom, as well as the left and right kidneys of the patient phantom. The images used to calculate these values were derived from simulations when the object-to-background ratios of 13:1 (Fig. 2a and b) and 6:1 (Fig. 2c and d) were used. The total counts obtained for each VOI were normalised to the maximum counts of the 3D OS-EM updates, obtained at 204 updates, and expressed as a percentage recovery. Similarly, the %RMS was normalised to the maximum percentage obtained at 204 updates.

As seen from Fig. 2a and c, fewer updates were required for the larger objects to reach convergence (count recovery > 90%). Convergence was reached for the right and the left kidney as well as the 65.4 ml sphere after only 24 updates. In contrast, the 14.1 ml and the 4.2 ml spheres achieved 90% recovery only after 48 and 84 updates, respectively. These observations applied to both concentration ratios. Figure 2b and d shows similar trends for the two concentration ratios where the %RMS increased rapidly by 32.5% for the 4.2 ml sphere from 24 updates to 84 updates. The 14.1 ml sphere had a %RMS increase of ≤ 31.1% from 24 updates to 84 updates. A smaller variation in the %RMS was observed for the larger objects, with a variation of ≤ 5.2% for the 65.4 ml sphere and ≤ 2.3% for the left and right kidneys.

#### Calibration factors

The results for the sphere-CF and the cylinder-CF were 13.9 cps/MBq and 16.6 cps/MBq, respectively, where the sphere-CF was less than the cylinder-CF by 16.3%. The results demonstrated that the VOI drawn based on the physical dimensions of the sphere excluded some of the counts for the sphere-CF due to spill-out. As a result, a lower CF value was obtained for the sphere-CF in comparison to that of the cylinder-CF.

#### Recovery coefficients

**Fig. 3:**
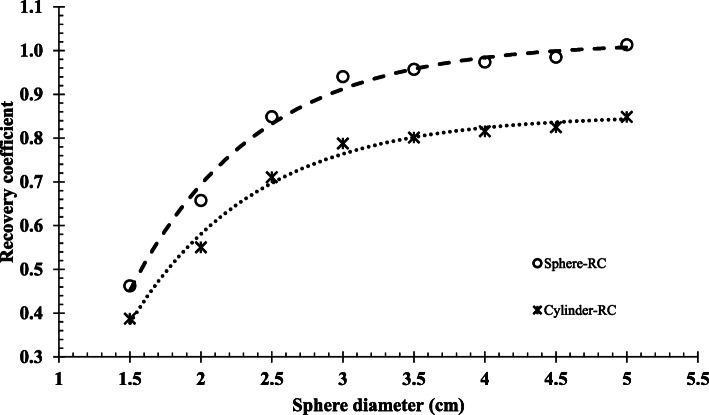
Recovery coefficient (RC) curves generated using a sphere calibration factor (CF) and a cylinder calibration factor (CF) plotted for different sphere sizes

Figure 3 shows the characteristic non-linear curves fitted with *R*^2^ values of 0.99 (Eq. 4), generated using the sphere-CF and cylinder-CF, when RC values were plotted for the eight different sphere sizes, allowing for interpolation between the sphere sizes. The curves provided the fraction of ^177^Lu activity concentration recovered from the reconstructed images for the given sphere sizes. In so doing, it allowed for the true concentration to be calculated, thus compensating for PVE. Although the simulated concentrations (*C*_true_) were the same for all VOIs, the smaller spheres had a lower SPECT estimated concentration (*C*_SPECT_) values, demonstrating the PVE. It is evident from these curves that RC is strongly dependent on the size of the spheres. The fit of the curves in Fig. 3 was used to determine the RC values of the four spheres, shown in Table 1, which were quantified in the three phantoms.

**Table 1:** The recovery coefficient (RC) values for the sphere and kidney volumes generated for the sphere and cylinder calibration factors (CFs)

Objects	Sphere-RC	Cylinder-RC	% difference
Kidneys			
L-K: 159 ml	1.18	0.99	16.1
R-K: 169 ml	1.18	0.99	16.1
Spheres			
15.6 ml	0.92	0.77	16.3
24.4 ml	0.96	0.81	15.6.
33.5 ml	0.98	0.82	16.3
65.4 ml	1.00	0.84	16.0
		Average	16.1 ± 0.26

*L*-*K* Left kidney, *R*-*K* Right kidney, *RC* Recovery coefficient

The sphere-RC and cylinder-RC for the spheres and kidneys are summarised in Table 1. The RC values ranged from 0.77 to 1.00 as the size of the sphere increased from 15.6 to 65.4 ml. The kidney RC values were 1.18 and 0.99 for the sphere-RC and cylinder-RC, respectively. Table 1 demonstrates a difference between the sphere-RC and the cylinder-RC values for all the simulated objects. The average sphere-RC value overestimated the average cylinder-RC value by 16.1 ± 0.26%, which may be attributed to the CF difference reported in the ‘Calibration factors’ section.

### Evaluation of quantification accuracy

#### Cylindrical phantom

Table 2 shows the quantification error results for the spheres simulated in the cylindrical phantom with and without PVC. The results were calculated using the sphere-CF and cylinder-CF without PVC, while the combinations of sphere-CF-RC and cylinder-CF-RC were applied to compensate for the PVE.

**Table 2:** Quantification error results for spheres simulated in a cylindrical phantom with and without partial volume corrections (PVCs)

Object	Quantification error (%)			
	Without PVC	With PVC	Without PVC	With PVC
Sphere	Sphere-CF	Sphere-CF-RC	Cylinder-CF	Cylinder-CF-RC
15.6 ml	− 4.56	3.32	− 20.08	3.28
24.4 ml	− 3.40	0.19	− 19.11	0.19
33.5 ml	− 2.05	− 0.43	− 17.98	− 0.43
65.4 ml	1.60	0.83	− 14.93	0.86
**Average**	− **2.10** ± **2.67**	**0.98** ± **1.64**	− **18.03** ± **2.23**	**0.98** ± **1.62**

*CF* Calibration factor; *PVC* Partial volume correction, *RC* Recovery coefficient

As seen in Table 2, the trend for the quantification error without PVC was as expected. The smallest sphere showed the largest quantification error, demonstrating the influence of PVEs, which was less important with increasing sphere size. The average quantification error obtained with the sphere-CF without PVC was − 2.10 ± 2.67% in comparison to − 18.03 ± 2.23% obtained with the cylinder-CF. The use of the cylinder-CF without PVC underestimated the true activity evidently for all the sphere sizes. PVC improved the average quantification error dramatically from − 18.03 ± 2.23 to 0.98 ± 1.62% for the cylinder results. The precision (standard deviation) of the average quantification error improved slightly with PVC for both the sphere and cylinder data. A slight overestimation (≤ 3.32%) of the activity values was observed for both phantoms for the smallest spheres when PVC was applied. The use of the sphere-CF-RC and cylinder-CF-RC resulted in comparable average quantification errors of 0.98 ± 1.64% and 0.98 ± 1.62%, respectively.

#### Torso phantom

The quantitative error results obtained for the spheres simulated in the torso phantom are demonstrated in Table 3. Similar to the cylindrical phantom, the results were analysed using the sphere-CF and cylinder-CF without PVC, and the corresponding sphere-RC and cylinder-RC were applied to correct for PVE.

**Table 3:** Quantification error results for spheres simulated in the torso phantom with and without partial volume corrections (PVCs)

Object	Quantification error (%)			
	Without PVC	With PVC	Without PVC	With PVC
Sphere	Sphere-CF	Sphere-CF-RC	Cylinder-CF	Cylinder-CF-RC
15.6 ml	− 4.92	2.92	− 20.39	2.99
24.4 ml	− 5.54	− 2.03	− 20.90	− 2.03
33.5 ml	− 4.84	− 3.26	− 20.32	− 3.26
65.4 ml	− 2.70	− 3.44	− 18.53	− 3.41
**Average**	− **4.50** ± **1.24**	− **1.45** ± **2.98**	− **20.04** ± **1.04**	− **1.43** ± **3.01**

*CF* Calibration factor; *PVC* Partial volume correction, *RC* Recovery coefficient

Without PVC, the quantification error showed an average underestimation of 4.50 ± 1.24% for the sphere-CF (Table 3). Similar to the sphere quantification results in the cylindrical phantom, the cylinder-CF considerably underestimated the quantified concentration, with an absolute average of 20.04 ± 1.04% for all sphere sizes.

It can be seen from the results in Table 2 and Table 3 that the calculated concentration underestimated the true concentration to the same extent between the two phantoms, with the worst quantification results obtained when applying the cylinder-CF with no PVC. Partial volume correction for both the sphere and cylinder slightly overestimated the concentration of the two smallest spheres (≤ 2.99%). Analogous to the sphere quantification in the cylindrical phantom, the application of the sphere-CF-RC and cylinder-CF-RC resulted in comparable average quantification errors of − 1.45 ± 2.98% and − 1.43 ± 3.01%, respectively.

#### Patient phantom

**Fig. 4:**
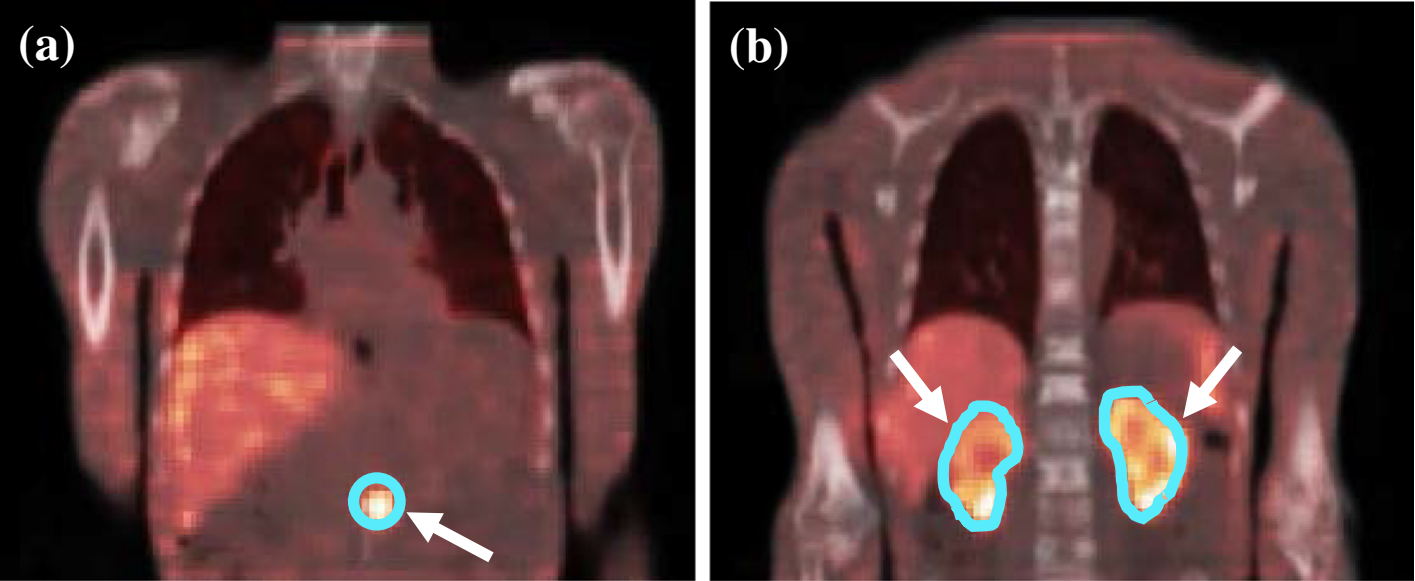
Reconstructed coronal slices of the patient phantom illustrating the **a** sphere and **b** kidneys used to determine the quantification error

Figure 4 shows the patient phantom’s reconstructed SPECT data with its associated CT data illustrating the sphere and the kidney objects (indicated by the arrows) used for quantification analysis. Table 4 compares the quantification errors obtained for the spherical and kidney objects simulated in the patient phantom calculated using the sphere and the cylinder data.

**Table 4:** Quantification error results for the spherical and kidney objects simulated in the patient phantom calculated with and without partial volume corrections (PVCs)

Objects	Quantification error (%)			
	Without PVC	With PVC	Without PVC	With PVC
Spheres	Sphere-CF	Sphere-CF-RC	Cylinder-CF	Cylinder-CF-RC
15.6 ml	− 4.42	3.47	− 19.97	3.53
24.4 ml	− 1.70	1.95	− 17.69	1.94
33.5 ml	− 0.34	1.32	− 16.65	1.32
65.4 ml	2.73	1.96	− 13.98	1.99
**Average**	− **0.93** ± **2.97**	**2.18** ± **0.91**	− **17.07** ± **2.48**	**2.20** ± **0.94**
Kidneys				
L-K: 159 ml	18.98	1.01	− 0.37	0.94
R-K: 169 ml	22.34	3.50	2.44	3.45
**Average**	**20.66** ± **2.38**	**2.26** ± **1.76**	**1.04** ± **1.99**	**2.20** ± **1.77**

*CF* Calibration factor, *L*-*K* Left kidney, *PVC* Partial volume correction, *R*-*K* Right kidney, *RC* Recovery coefficient

As seen from Table 4, the sphere quantification errors showed similar trends to those obtained in the cylindrical and torso phantoms. A small average quantification error of − 0.93 ± 2.97% was obtained when applying the sphere-CF without PVC in the patient phantom. Partial volume correction using the sphere-CF-RC altered the average quantification error to 2.18 ± 0.91%. These results were comparable to those obtained in the torso (− 1.45 ± 2.98%) and the cylindrical phantom (0.98 ± 1.64%). The cylinder-CF underestimated the absolute average quantified concentration of the spheres (17.07 ± 2.48%) similar to the torso (20.04 ± 1.04%) and the cylindrical phantom (18.03 ± 2.23%). Partial volume correction improved the cylinder-CF-RC to an average of 2.20 ± 0.94% similar to the torso (− 1.43 ± 3.01%) and cylindrical phantom (0.98 ± 1.62%). When all corrections were applied, the quantification accuracy using the sphere-CF-RC (2.18 ± 0.91%) was comparable to that of the cylinder-CF-RC (2.20 ± 0.94%) data, validating the quantification accuracy findings of the cylindrical and torso phantoms.

The kidney quantification showed an average overestimation of 20.66 ± 2.38% for the sphere-CF without PVC compared to the better results (1.04 ± 1.99%**)** obtained for the cylinder-RC. This was contrary to the sphere quantification results where a considerable underestimation of the quantification error was obtained using the cylinder-CF without PVC. The kidney quantification error improved to 2.26 ± 1.76% with the sphere-CF-RC corrections. With PVC the sphere-CF-RC and cylinder-CF-RC yielded comparable average quantification results of 2.26 ± 1.76% and 2.20 ± 1.77%, respectively; similar to the findings of the sphere quantification results. The results suggest that PVC did not play a major role in the kidney quantification error using the cylinder data. This may be attributed to the fact that the cylinder-RC value applied to the kidneys was close to 1.0.

## Discussion

### Quantification steps

#### 3D OS-EM optimization

The trends observed in Fig. 2a and c were similar, and convergence (recovery ≥ 90%) was observed at 84 updates for all the objects indicated by the 90% dashed line. Although better reconstruction accuracy, beyond the 90% recovery, could be reached at higher updates, the following was taken into consideration. A higher number of updates increased the number of recovered counts, but has also been shown in the literature to increase image noise levels [27, 60]. This was also confirmed in our study when considering the graphs shown in Fig. 2b and d. In addition, the use of CDR compensation in the reconstruction process has been shown to result in pronounced edge ringing artefacts with an increased number of iterations, particularly for larger objects [61, 62]. The larger increase (32.5%) in the %RMS for the 4.2 ml sphere (Fig. 2b and d) in comparison to the smaller variation (≤ 5.2%) for the largest objects (65.4 ml sphere, the left and right kidneys) was expected due to the lower mean value obtained for the smaller sphere owing to the PVEs. Leong et al. [29] reported similar trends in comparative investigations. Furthermore, the reconstruction computational times are longer with increased updates, which is an important consideration for practical implementation. The data presented in Fig. 2a and c shows that the number of updates used was governed by the smallest sphere (4.2 ml), which only reached the 90% recovery at 84 updates. Although there is a larger increase in the %RMS for the smallest sphere from 24 updates to 84 updates, choosing updates less than 84 updates would compromise the recovered counts and the recovered activity. It was stated by Dewaraja et al. [27] that a large number of iterations is justified to obtain accurate quantitative information in post-therapy administrations where noise is not a significant problem. Our study’s findings were on par with Brolin et al. [52] and Ljungberg et al. [16], who used a total of 80 OS-EM updates for quantification of ^177^Lu-DOTATATE distribution. Therefore, 84 updates were considered sufficient to attain a 90% recovery for all investigated object sizes used in our study and were further used for reconstruction of the SPECT data.

#### Calibration factors

The underestimation of the sphere-CF in comparison to the cylinder-CF value was attributed to PVE due to spill-out of counts. Some authors have suggested the simple use of an additional 3 cm margin to the physical dimensions of the sphere to include spilled-out counts [42]. Other authors have defined VOIs according to the physical dimensions of the CF sphere [63] or bottles [15] for quantification of ^177^Lu SPECT studies. We found purpose in defining the sphere-CF VOI as the physical dimensions of the sphere in order to have a consistent delineation method for the quantified spheres. The 65.4 ml sphere was chosen to limit PVEs; however, the comparative assessment between the cylinder-CF and the sphere-CF indicated that the effect of spill-out counts from the sphere was not eliminated but was reduced. The cylinder VOI was selected centrally on the cylinder image where the effects of spill-in and spill-out of counts were presumed to cancel. Several ^177^Lu SPECT CFs, obtained for the Siemens Symbia series (Siemens, Healthineers, Germany) gamma cameras, similar to that simulated in the current study, have been reported in the literature. The reported CFs were all obtained for a 0.95-cm-thick NaI (Tl) detector crystal equipped with a ME collimator using a 20% energy window centred over the 208 keV photopeak. The reconstructed images were all corrected for attenuation, scatter and CDR; however, the correction methodologies varied. Although the CFs were acquired with similar gamma camera settings, the values differ vastly depending on the phantom configurations, VOI definition, the gamma camera used, and the types of corrections applied to the data. A value of 20.2 cps/MBq for a cylindrical phantom with a VOI containing the entire phantom is reported for a Siemens Symbia Intevo Bold SPECT/CT system (Siemens Healthineers, Germany) [32]. The same authors reported values of 18.86 cps/MBq and 20.36 cps/MBq for VOIs drawn centrally in the cylindrical phantom and encompassing the entire phantom [64]. While other authors have reported values of 10.1 cps/MBq and 10.3 cps/MBq for the Symbia T16 (Siemens Healthineers, Germany) obtained using a cylindrical phantom and by applying a 50% threshold of the maximum voxel value to define the VOI for the CF [65]. Mean CF values (simulated for different gamma cameras) of 10.5 cps/MBq, 9.5 cps/MBq and 10.1 cps/MBq, for phantom geometries, which included a sphere in a non-radioactive background, a sphere in a radioactive background and a uniform cylindrical phantom, respectively, have been reported [43]. The above variations illustrate the variability obtained with the different gamma cameras, processing software, phantom geometries and VOI definitions, emphasising the need to establish a CF suitable for a specific SPECT quantification study.

#### Recovery coefficients

Creating sphere-RC and cylinder-RC curves aimed to obtain approximate RC values for spherical objects of different sizes where spill-out of counts were present and when PVC is required. In order to use sphere-RC and cylinder-RC curves from Fig. 3 for PVC, the physical size of the sphere is needed and may be obtained from the CT image of the study. The difference between the sphere-RC and the cylinder-RC was demonstrated by the average difference of 16.1 ± 0.26%. The results were consistent for both the spheres and the kidneys. The discrepancy was attributed to the differences between the two CFs used to calculate the RCs. The overestimation of the sphere-RC served as compensation for underestimating the sphere-CF and vice versa for the cylinder-RC and cylinder-CF. The cylinder-RC value of 0.81 for the 24.4 ml sphere was comparable to the 0.85 RC value reported by Hippeläinen et al. [15] for a 26 ml sphere. Similarly, Sanders et al. [63] reported a RC value of 0.80 for a 16 ml sphere compared to our 0.77 value for the same size sphere. Our study contained spherical objects mimicking tumours, and therefore PVC using curves from Fig. 3 may be applicable if the object in the phantom has a similar configuration. The RCs may differ if the object’s shape has an irregular form, or a non-uniform activity distribution [32]. For this reason, the kidney RC values were calculated directly using Eq. 3 and not from the fitted RC curves. This method is applicable when the size and activity concentration of the object is known. The size of the objects may be obtained from high-resolution images such as CT or MRI.

### Evaluation of quantification accuracy

The sphere quantification error results (Table 2 to Table 4) obtained with the sphere and cylinder CF and RC data showed similar trends for all three phantoms. The slight overestimation of the activity values observed for the smallest sphere (15.6 ml) when PVC was applied for both the sphere and cylinder data may be attributed to the discrepancy between the calculated RC value and that obtained from the fitted function shown in Fig. 3. It can be seen from Fig. 3 that the function slightly underestimated the calculated sphere-RC and cylinder-RC values at a sphere diameter of 3 cm, comparable to the diameter of the smallest quantified sphere (3.1 cm). Another source for this discrepancy may be found in the different object-to-background ratios used for the RCs compared to those used to assess the quantification error. A different object-to-background ratio was selected to avoid bias in the quantification evaluation of the phantoms. The sphere-CF without PVC showed a general tendency of a small quantification error for the quantified spheres compared to the large underestimation by the cylinder-CF. This may be expected due to the similar geometry between the quantified spheres and the sphere used for the sphere-CF. These results illustrated the dependence of the quantified error on the geometry of the CF. Whole-organ specific CFs, obtained using 3D-printed phantom inserts in the shape of the spleen, kidney, pancreas and liver, have been shown to improve the accuracy of organ dosimetry for ^99m^Tc and ^177^Lu SPECT studies [55]. This type of dependence was verified in our study by extending the quantification error evaluation to a clinical kidney geometry. Without PVC, a large overestimation of the quantified kidney concentration for the sphere-CF was obtained vs. the small kidney quantification error obtained with the cylinder-CF. The total counts used to determine the kidney quantification error were equivalent when using the sphere-CF or the cylinder-CF. Therefore, the large overestimation may be explained, at least in part, by the VOI selection for the sphere-CF and cylinder-CF, which resulted in differences in the contribution of spill-in and spill-out of counts due to the PVE. The lower value of the sphere-CF, compared to the cylinder-CF, demonstrated this effect. This CF discrepancy may also offer a possible explanation for the difference in the kidney quantification trend found between the current study and other literature studies that reported underestimations of kidney activity without PVC [16, 32]. These studies applied a ‘nearly partial-volume-free CF’ determined from a cylinder that was less susceptible to spill-out effects [32]. Another important consideration is that the kidneys used in our study were segmented and delineated as whole kidneys without separate compartments for the cortex, medulla and renal pelvis areas. This will affect the shape of the kidneys and, as a result, the corresponding PVEs. The discrepancy may presumably be exacerbated by the fact that the kidney RCs were calculated directly from Eq. 3 instead of using a look-up curve or table. This may lead to some bias in the quantification results. Therefore, it is important to note both the geometry and the delineation method used for the CF, RCs and object of interest. The different effects of the two CFs, used in our study, were balanced by their corresponding RC values. This was shown by the comparable average quantification error results obtained for the different-sized spheres in all three phantoms as well as the kidneys in the patient phantom.

The quantification errors obtained in this study compared well with literature investigations of similar phantom and patient geometries, which employed OS-EM-based reconstruction algorithms with compensation for scatter, attenuation using CT data and CDR. De Nijs et al. [58] investigated the ^177^Lu SPECT activity quantification of radioactive spherical inserts placed in a cylindrical phantom with a radioactive background. The authors applied the ESSE scatter correction method and compensated for PVEs by drawing VOIs larger than the spherical inserts and reported errors of 10% for their largest sphere of 37 ml. Sanders et al. [63] reported larger errors of up to 20% for smaller sphere inserts (16 ml) in the same geometry, using the TEW scatter correction and VOIs defined according to CT data without PVC. An average percentage error of 6.6 ± 3.5% for quantification of 175 ml cylindrical inserts placed in a larger cylinder was reported by Beauregard et al. [49]. The authors used the DEW scatter correction and manually adjusted the VOIs using a percentage threshold of maximum activity to compensate for spill-out due to PVEs. Therefore, the quantification errors (≤ 0.98 ± 1.64%) found for the cylindrical phantom used in our study were satisfactory when all corrections were applied.

Hippeläinen et al. [15] investigated the ^177^Lu SPECT activity quantification of spheres measured in the same torso RSD phantom, as simulated in our study, and reported errors of 15% for their largest spherical insert (104 ml). They used an accelerated MC simulation method for scatter correction and CT-based VOIs without PVC. The quantification errors found in our study (≤ − 1.43 ± 3.01%) were comparable to that of D’Arienzo et al. [66] who quantified a cylindrical insert (19.13 ml) in an anthropomorphic torso phantom (Data Spectrum Corporation, USA) with an accuracy of 2.0%. D’Arienzo et al. [66] applied a transmission-depended convolution subtraction scatter compensation method and RCs, as defined in the current study, for PVC. Uribe et al. [57] used a thorax phantom (Data Spectrum Corporation, USA) and reported accuracies of better than 5.0%, for their largest insert (34 ml) using the analytical photon distribution interpolated for scatter correction. The accuracy was achieved for their iterative adaptive dual threshold segmentation method, which recovered the activity better than the CT-based and 40% fixed threshold segmentation methods. Both these methods underestimated the activity due to PVEs, grossly.

Beauregard et al. [49] reported quantification error results of 2.6 ± 1.8% for ^177^Lu SPECT activity data of five patients acquired with the Symbia TruePoint T6 SPECT/CT gamma camera (Siemens, Healthineers, Germany). The authors evaluated their quantification error by comparing the calibrated activity injected into the patients with the quantified activity obtained by drawing a VOI surrounding the patient using a − 400 Hounsfield unit threshold for the CT images. Although their quantification method was different from ours, their results were comparable to our patient phantom findings, when all corrections were applied, with sphere and kidney quantification errors of ≤ 2.20 ± 0.94% and ≤ 2.26 ± 1.76%, respectively. Sanders et al. [63] compared ^177^Lu bladder activity concentrations obtained from SPECT/CT patient images with urine sample measurements from calibrated well counters. Their segmentation method for the bladder included placing a smaller ellipsoid VOI in the bladder’s interior to avoid edge roll-off that causes variations in the iso-contour measurements. The total mean counts in the VOI were normalised to the volume of the bladder. The method yielded mean quantification errors of 10 ± 8.3%. Bailey et al. [14] reported an accuracy of ± 10% for ^177^Lu activity quantification of whole body planar scans and cardiac blood pool SPECT images. Hippeläinen et al. [15] reported errors of up to 25% for kidney quantification of patients who underwent ^177^Lu-DOTATATE treatment using SPECT images. The systematically higher activity quantification errors were attributed to CT-based VOIs that were sensitive to PVEs and estimates obtained at different time points. Willowson et al. [56] investigated the accuracy of using a single time point for renal dosimetry and showed improvement in the quantification accuracy to 13% and 2% when using the 4-h and 24-h data only. Similarly, the kidney volumes were defined on the co-registered CT data; however, no information on PVC was available. Optimization of kidney quantification for ^177^Lu SPECT/CT using geometry specific RCs for PVC was investigated using a 3D-printed two-compartment kidney phantom [32]. The best quantification accuracies of 1.5% and 10.3% were reported using the commercially available reconstruction algorithms xSPECT and Flash-3D (Siemens, Healthineers, Germany), respectively, when model-based RCs were applied to compensate for PVE. These studies demonstrate the disparity in quantitative accuracy found for the different imaging geometries processing protocols and methods evaluating the quantitative error. The overall improved quantitative results obtained in our study when all corrections are applied may be indicative of the accuracy of the correction methods used in the reconstruction process.

## Conclusion

In this study, we used the SIMIND MC program to model a Siemens Symbia T16 gamma camera for ^177^Lu SPECT imaging of voxelized phantoms as validated by Ramonaheng et al. [47]. Emanating from the fact that there is currently no consensus on the ideal SPECT CF geometry for quantitative data, we presented two CFs and their corresponding RCs. The quantification errors obtained with the two-combination sphere-CF-RC and cylinder-CF-RC were evaluated by quantifying different size spheres in three different phantom geometries as well as the kidneys in the patient phantom. Our study showed the effect on the quantification error, by using the sphere-CF or the cylinder-CF, without PVC, depends on the quantified geometry. The quantification results emphasised the importance of applying a PVC with an RC obtained with the same CF used to convert the quantified data into units of concentration. We demonstrated that when all corrections were applied (attenuation, scatter, CDR and partial volume), the ^177^Lu SPECT quantification errors in the three phantoms were comparable for the sphere-CF-RC and cylinder-CF-RC combinations. Our absolute quantification errors of smaller than and equal to 3.53% for the three phantoms, compared well to literature findings and complied with the ± 5% absorbed dose accuracy required for molecular radiotherapy [67]. Although our findings suggest the feasibility of obtaining accurate ^177^Lu SPECT activity quantification upon the careful selection of a CF-RC combination, certain considerations may be limiting. These include firstly, that the application of patient-specific RCs in the clinic entails the use of CT or MRI data in combination with MC simulations. This would require modelling of CF-RC combinations for every patient geometry and activity distribution, which may be cumbersome to implement routinely. Secondly, the presence of non-uniform activity distributions may further complicate activity quantification. Thirdly, the segmented kidney volumes used in this study were obtained from low-dose, low-resolution, non-contrast-enhanced CT data, which was inadequate to differentiate between the different kidney compartments, such as the medulla, cortex and renal pelvis, reliably. For this reason, each whole left and right kidney was assigned a uniform activity concentration. High-resolution image data such as contrast-enhanced CT images or MRI would facilitate segmentation and VOI delineation that is more reliable with a better representation of a clinical kidney model. Another limitation of this study is the methodology used to calculate the kidney RCs directly from Eq. 3, which requires prior knowledge of the kidneys’ activity concentration, and is not clinically available. This study reinforces the need to standardise segmentation methods for CFs, RCs as well as tumour and organ delineation.

## Data Availability

The datasets generated during and/or analysed during the current study are available from the corresponding author on reasonable request.

## Abbreviations

CDR: Collimator detector response
CF: Calibration factor
cylinder-CF: Cylinder calibration factor
cylinder-RC: Cylinder recovery coefficient
cylinder-CF-RC: Cylinder calibration factor and cylinder recovery coefficient
C_SPECT_: SPECT estimated activity concentration
*C*_true_: True activity concentration
DEW: Dual energy window
ESSE: Effective source scatter estimation
FWHM: Full-width half-maximum
MIRD: Committee on medical internal radiation dose
MC: Monte carlo
ME: Medium-energy
NEMA: National electrical manufacturers association
OS-EM: Ordered subset expectation maximization
PRRT: Peptide receptor radionuclide therapy
PVC: Partial volume correction
PVEs: Partial volume effects
RSD: Radiological support devices
RCs: Recovery coefficients
RMS: Root mean square
SIMIND: Simulating medical imaging nuclear detectors
sphere-CF: Sphere calibration factor
sphere-RC: Sphere recovery coefficient
sphere-CF-RC: Sphere calibration factor and sphere recovery coefficient
TEW: Triple energy window
VOI: Volume of interest
